# Barriers and enablers to engagement in exercise and physical activity in non-English speaking South Asian people with chronic musculoskeletal disease

**DOI:** 10.1186/s41927-024-00372-z

**Published:** 2024-03-11

**Authors:** Nasimah Maricar, Gillian Yeowell, Trixy David, Behram Khan, Anne Barton, Kimme L Hyrich, Sandra E Hartley

**Affiliations:** 1 Northern Care Alliance NHS Foundation Trust, Salford Royal NHS Foundation Trust, Manchester, UK; 2 Centre for Epidemiology Versus Arthritis, Centre for Musculoskeletal Research, Division of Musculoskeletal and Dermatological Sciences, University of Manchester, Manchester, UK; 3 Department of Health Professions, Manchester Metropolitan University, Manchester, UK; 4 The Kellgren Centre for Rheumatology, Manchester University NHS Foundation Trust, Manchester, UK; 5 Centre for Genetics and Genomics Versus Arthritis, Centre for Musculoskeletal Research, Division of Musculoskeletal and Dermatological Sciences, University of Manchester, Manchester, UK; 6 NIHR Manchester Biomedical Research Centre, Manchester Foundation NHS Trust, Manchester Academic Health Science Centre, Manchester, UK

**Keywords:** Osteoarthritis, Inflammatory arthritis, Musculoskeletal pain, Activity, Fitness, Qualitative

## Abstract

**Background:**

Exercise and physical activity (EPA) are recommended for people with chronic musculoskeletal disease; however, lower levels of engagement with EPA has been consistently reported in people from the South Asian community across a range of diseases. As language can pose a significant barrier in healthcare, this study aimed to understand the enablers and barriers to the acceptance of EPA among non-English speaking South Asian people who attended rheumatology clinics.

**Methods:**

12 non-English speaking individuals from the South Asian community who had chronic musculoskeletal disease with significant pain scores were interviewed via telephone or face-to-face in their spoken languages. The audio recordings of the interviews were translated into English and transcribed verbatim. Data was analysed using thematic analysis implemented in the NVivo 12 Pro software program.

**Results:**

The mean age was 52 years (9 women and 2 men). One main theme was identified: ‘Enablers and barriers to exercise and physical activity’. Enablers to EPA were having knowledge about the benefits of EPA, being given resources in a language that they understood, and supportive environments such as having access to community facilities for those who could not undertake EPA in their houses. Barriers included physical health such as pain and fatigue, lack of time, difficulties with transportation to exercise venues, dislike of group exercises and lack of understanding of what and how to do exercise and be physically active. Participants’ beliefs about EPA and whether they impacted their physical health seemed to influence whether they were undertaken or not. There was a perception that their culture shaped their compatriots’ beliefs about EPA, and it was not normal practice for people from their country of birth to engage in it.

**Conclusions:**

This is the first qualitative study to explore the barriers and enablers to engagement in EPA in non-English speaking South Asian people with chronic musculoskeletal disease. Modifiable factors such as addressing the level of knowledge on the benefits of EPA in the management of chronic joint and muscle pain; aiding the development of the skills required to exercise safely and confidently despite chronic pain and providing information and services in the native language could promote the EPA engagement of non-English speaking South Asian individuals with chronic musculoskeletal disease. The findings may inform improvements within clinical services to promote the benefits, impact and self-efficacy of engagement with EPA as part of chronic musculoskeletal disease management.

**Ethics approval:**

The West Midlands-Edgbaston Research Ethics Committee (reference:20/WM/0305).

## Background

Many international guidelines including the American College of Rheumatology, European League Against Rheumatism (EULAR) and the National Institute for Health and Care Excellence (NICE) advocate exercise and physical activity (EPA) for the management of chronic musculoskeletal disease such as osteoarthritis, rheumatoid arthritis and spondyloarthritis [1–4]. A wide range of evidence-based exercise interventions such as walking, tai chi, strengthening, neuromuscular training and aquatic exercise, among others, have been shown to effectively improve pain and function for chronic musculoskeletal disease [1–4]. There is also strong evidence for the benefits of EPA on improving disease activity, in the prevention of comorbidities, in the management of chronic pain and in the promotion of better physical and mental health [5–8]. Whilst EULAR has proposed a list of general and disease-specific barriers for engagement with physical activity and facilitators that can aid people to be more physically active, these are focussed on Caucasian people with chronic musculoskeletal pain and resources are only available in the English language [4].

South Asian (SA) people represent the highest proportion of all ethnic minorities in the United Kingdom (UK) [9]. People of SA descent may include those who originate from India, Sri Lanka, Pakistan, Bangladesh or Nepal. Engagement with EPA among the SA community is reported to be significantly lower than the White majority population [10–18]. According to a recent systematic review that investigated EPA and sedentary behaviour among SA adults, only 53–61% of SA people met the World Health Organization recommendation [13, 17] of 150 min/week moderate to vigorous EPA [17] and recent UK government data reported 43.8–56.1% SA adults compared with 60.5–72.4% White people met the recommended guidelines [18]. This has significant implications in those SA individuals who may also have a chronic musculoskeletal disease since many clinical guidelines recommend exercise as an essential part of treatment. However, the lack of engagement with EPA has not previously been explored in SA people with chronic musculoskeletal disease [17].

Several barriers and enablers to EPA have been reported for SA adults without chronic musculoskeletal disease [10, 19, 20]. For example, one systematic review collated findings from a series of qualitative studies that explored barriers and facilitators to EPA uptake and adherence among SA older adults residing in Canada and the UK [19]. They found language barriers, support networks, beliefs of ill health resulting from ageing and fate, and environmental factors such as weather, safety and accessibility of exercise facilities were factors that could influence engagement with EPA [19]. Another systematic review, evaluating both qualitative and quantitative studies on EPA among SA immigrant women, found similar cultural and structural barriers to engagement with EPA, and reported faith and education could serve as facilitators [10].

Studies in the UK have reported that barriers to EPA can be further compounded by lack of fluency in English, which may restrict attendance and participation in EPA programmes [21–23]. Several studies have also reported that underserved populations in the UK and America, such as non-English speaking groups, have insufficient support to access or effectively engage in EPA [23–26]. As language could pose a significant barrier in healthcare, the aim of this study was to seek an understanding of the barriers and enablers to engagement with EPA among SA people with painful chronic musculoskeletal disease accessing healthcare under the National Health Service (NHS) in the UK who had the additional language disadvantage of being non-English speaking.

## Methods

The study is reported in accordance with the consolidated criteria for reporting qualitative (COREQ) research [27]. A qualitative study [28], underpinned by an interpretive paradigm [29] was undertaken to investigate the research aim. This involved one to one semi-structured interviews with participants.

### Study population

A purposive sample was used to recruit non-English speaking SA adults aged 18 years or older, who attended a rheumatology clinic in one NHS trust in Northwest England and who met the eligibility criteria including having significant chronic musculoskeletal pain defined as a score of 5 or above on the Visual Analogue Scale (VAS) (Table 1). The main exclusion criteria ensured participants had no conditions that would render EPA unsafe for them (Table 1). Recruitment continued until data saturation was achieved [30, 31].

**Table 1:** Eligibility Criteria

Inclusion Criteria	Exclusion Criteria
• Non-English speaking South Asian including those who are not fluent in English and requiring interpreting services • Adults ≥ 18 years • Capable of giving informed consent • Have a physician’s diagnosis associated with chronic pain, including but not limited to chronic widespread pain, fibromyalgia, osteoarthritis and inflammatory arthritis • Pain scoring of 5 or above on the Visual Analogue Scale (VAS)	• Learning disabilities that will impede understanding of the study and the ability to give informed consent • Speech or cognitive impairment including from neurological diseases and previous stroke • Severe comorbidity that prevents the ability to engage with exercises or physical activities such as unstable heart diseases, severe chronic obstructive pulmonary diseases, unstable fractures • Recent joint replacement surgeries, recent fractures and impending surgery that can affect engagement in exercise and physical activity • Fluent in English, that is, having an understanding of spoken and written English and ability to speak, read and write English

### Data collection

Following written informed consent for those seen face to face in clinic and electronically signed telephone informed consent for those seen during virtual consultations, participants were invited to take part in an in-depth one-to-one interview that took place at their chosen time. Each interview lasted between 30 and 60 min. The interviews were undertaken by two of the research team (TD, BK), who were rheumatologists of SA origin, who spoke the participant’s language. Having interviewers from a SA background was deemed helpful not only to promote rapport between the interviewers and the participants but also because of their awareness of cultural sensitivities. An interview guide was used to explore themes relating to the aims of the study. The interview guide was developed following a review of the literature and consultation with local stakeholders (members of the public and general practitioners) during a patient and public involvement and engagement event. The guide was piloted in a rheumatology clinic with two patients by the same interviewers leading to minor refinement of the syntax of the language.

The interview guide was used to direct the interview; however, further discussion was guided by the participant’s response to the questions to ensure sufficient flexibility to allow new topics to be fully explored. Interviews were digitally audio recorded.

### Data analysis

The audio recordings of the interviews were translated into English and transcribed verbatim by a local transcriber. As the transcriber was part of the research process, the transcriber was selected to be of SA heritage, working within the healthcare profession with some knowledge of chronic musculoskeletal disease [32–34]. All transcripts were read and cross-checked by the two interviewers to ensure accuracy of the translation. The data was analysed using thematic analysis adopting the framework described by Braun and Clarke and implemented in the NVivo 12 Pro software program [35]. This involved an iterative process of coding and constant comparison to map and link themes into basic, organizing and global themes [36]. Data is available on request.

## Results

Of 13 people invited, 12 individuals consented and attended one-to-one interviews in their spoken languages: Urdu, Punjabi or Hindi. All were telephone interviews except one conducted face-to-face. The mean age was 52 years (range: 40–68; SD: 9.5). The participants’ characteristics are summarised in Table 2.

**Table 2:** Participants’ characteristics

Code	Gender	Age (years)	Country of origin	Spoken languages	Marital Status	Religion	No. of children	No. of households (including children)	Highest Educational level	Employment	VAS (0–10)	Co-morbidities
A1	Female	44	Pakistan	Urdu	Married	Islam	3	5	GCSE	Housewife	5	Graves’ disease, T2DM, RA, possible psoriasis
A2	Female	41	Pakistan	Urdu	Married	Islam	2	4	GCSE	Housewife	7	Dermatomyositis, T2DM, previous MI
A3	Female	54	Pakistan	Urdu/ Punjabi	Married	Islam	0	2	Primary	Housewife	10	RA, GI issues
A4	Female	55	Pakistan	Urdu	Married	Islam	1	3	GCSE	Housewife	7	MCTD, Raynaud’s phenomenon
A5	Female	49	Pakistan	Urdu	Married	Islam	3	5	Primary	Housewife	10	CTD/Sjogren’s, chronic pain
A6	Female	49	Pakistan	Urdu	Married	Islam	3	4	Postgraduate masters	Unemployed	8	skin vasculitis, fibromyalgia
A7	Female	55	Pakistan	Urdu/ Punjabi	Married	Islam	1	2	Secondary	Housewife	10	inflammatory arthritis, hypertension, hyperlipidaemia, T2DM, hypothyroidism, vitiligo
A8	Female	63	Pakistan	Urdu	Married	Islam	4	3	Primary	Housewife	10	RA, fibromyalgia, OA
A9	Female	68	Pakistan	Urdu	Single	Islam	0	4	GCSE	Unemployed	10	RA
A10	Male	66	Pakistan	Urdu	Divorced	Islam	0	1	GCSE	Unemployed	10	previous diagnosis of mild SLE/MCTD, OA, facial telangiectasia, cataracts, hypertension, asthma
A11	Male	45	Bangladesh	Urdu/ Bengali	Married	Islam	4	6	12th grade	Employed	6	RA, bilateral CTS
A12	Male	40	India	Hindi	Married	Hindu	2	4	Secondary	Employed	10	chronic back pain

Index: RA– rheumatoid arthritis; T2DM– type II diabetes; MI– myocardial infarction; GI– gastrointestinal; MCTD - mixed connective tissue disease; CTD - connective tissue disease; OA– osteoarthritis; SLE - systemic lupus erythematosus; CTS– carpal tunnel syndrome

**Fig. 1:**
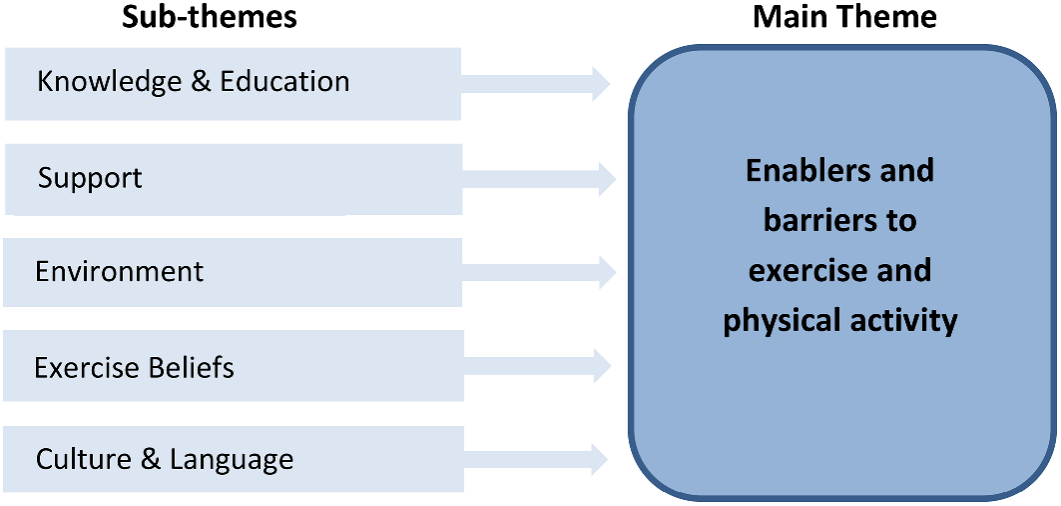
Key themes and sub-themes

Analysis of the data confirmed data saturation had been achieved. One overarching theme was identified: ‘Enablers and barriers to exercise and physical activity’, with several associated sub-themes common to both (Fig. 1). Therefore, data regarding enablers and barriers to EPA have been presented under each sub-theme. Pseudoanonymised verbatim quotes have been used to support each sub-theme.

### Theme: Enablers and barriers to exercise and physical activity

#### Knowledge & education

There appeared to be many barriers and enablers to participating in EPA that were highlighted by participants. There was a perception, by some, that the lack of engagement in EPA was because many had not been educated about EPA and therefore did not know about the positive effects on their health.

*I don’t think they know much about the reasons for exercise. I think they should be told about the benefits, and they shouldn’t ignore this. (A2)*

*Yes, if somebody told me to do exercise like this, then I would have had some hope. Can I say something that someone can be educated and someone can not be educated and someone can be lazy but I’m not lazy. If someone could have told me and explained to me and showed respect and maybe ages before, I could have got better. (A5)*

This was reinforced by other participants whose lack of awareness of EPA and how they could undertake the activity seemed to provide a barrier to their participation with it. Helping participants to become more informed about EPA could therefore help to facilitate their engagement with physical activity.

*I’ve not done lots of exercise. I’ve not got much of an idea of this. If I don’t exercise at all, maybe then I would realise that by exercising, it is better for me. (A3)*

*If there were such activities for us patients where we can go and get support to do the exercise. Rather than eating medicine and laying down. Support to get us active. (A6)*

It was therefore surprising that many participants had not been referred to physiotherapy even though there appeared to be a willingness to go. For those who did get the opportunity, for some, it provided the chance to gain knowledge about EPA, which was seen to facilitate their engagement with it.

*That would be very good [attending physiotherapy] because I don’t really understand what exercise to do. I may just move my leg around here or there. I try and do certain things with my hands. (A9)*

*When I had physio, they taught me some exercises in a good way.… I couldn’t climb the stairs and the physio taught me how to take one step at a time to climb the stairs and put weight on my arm. They taught me a lot in their way. (A2)*

However, it was apparent that not everyone got a benefit from attending physiotherapy.

*I feel that the physiotherapy doesn’t make much difference…. [I was told] sit like this, move your legs like this. It doesn’t really benefit me, whilst with walking I feel a bit better… (A11)*.

*They gave me a book to sit down and do arm exercises. I try but I can’t do it because I have pain in my arms, fingers and shoulder. This is a major difficulty. (A6)*

*I have been there [to physio]. I said I can’t do that (A8)*

Additionally, for some there appeared to be a lack of understanding as to what EPA was. One participant communicated that they were in too much pain to undertake EPA as it made their pain worse, yet they spoke favourably about swimming, which they did not perceive to be EPA.

*Yes, it makes you feel good [swimming]. Your body gets tired. I feel I…. get a good sleep, otherwise I don’t get much sleep. (A12)*

There was also confusion about whether activities of daily living counted towards their

*You mentioned housework, I’ve never really classed that as exercise. (A10)*

*We think we do a bit of cleaning or washing, and we think that’s exercise. (A9)*

### Support

Some participants acknowledged that they had been given resources to support their engagement in EPA, such as booklets or information sheets. However, what seemed to be important was that the resources that they were provided with were in a language that they understood to maximise their benefit.

*If it’s explained in Urdu, then it’s better because I can understand everything then. In English, I try to understand some of the body language because there’s some words that I can’t understand. (A2)*

*If it’s a leaflet, I’d prefer Bengali. (A11)*

*… if it was in Hindi that would be better (A12).*

Nevertheless. not everyone wanted to be educated in this way or fully benefitted from having such resources.

*I would just like to say, I’m not really interested in the booklets. I don’t even feel like looking at them. (A6)*

Several participants were more motivated to engage in EPA if they were supported by other people. Family or friends in particular appeared to be influential in fostering this undertaking.

*Even if I feel I can’t walk, my children give me encouragement and that helps me to start walking. (A2)*

*Both my sons are studying medicine, so they continuously tell me to exercise and it’s good for you.(A11)*

*He [husband] says to go out more but I can’t walk that much. He takes me to the park for a short while, but I can’t walk very far. I can’t go everyday. Maybe once a week, I can go. (A3)*

Undertaking EPA in a group with other people was highlighted by some to be more motivating to support their engagement with the activities.

*If you exercise at home alone, you may do it for one day or two days. Whereas with combined, you can speak to someone, see them and you can exercise at the same time. And you don’t realise the time and you get your exercise done too and you can talk and have a conversation at the same time. (A1)*

*If they gave activities to do and when you see other people, it gives you encouragement. It changes your mind when you look at other people. (A6)*

This seemed particularly the case if the group catered specifically to their needs, which for some women, meant being in same-sex groups.

*If it’s [just] ladies, then you feel more comfortable. (A6)*

*Ladies only [is better] (A4)*

However, for others same sex groups was not a priority.

*It’s no problem [mixed groups]. Because you have to think about what’s best for you. These small things don’t matter. (A2)*

*I think if it’s ladies, it’s better, but if there’s gents then it’s o.k (A1)*

Group activities, though, were not the preference of everyone. Lack of time to engage in this way could provide a barrier to the undertaking of EPA.

*If I was to go to a class, I would have to take out the time to go. At home, I can exercise in the garden. There’s no time limit. It could be during the day or at night-time. (A11)*

### Environment

The environment also seemed to influence the ability to be active. Having a garden and enough space in the home appeared to provide more opportunities for participants to engage in EPA.

*For exercise, I’ve got a room in the garden. It’s like a gym and I’ve got a running machine in there. I’ve got space in the house to exercise as well. (A11)*

*I do some gardening, this way I get some physical exercise and also my mind stays fresh. (A2)*

Walking was one of the most popular activities that the participants undertook. Having a park nearby and somewhere to rest whilst out facilitated some participants’ engagement in EPA as it supported their ability to walk.

*Everyone has their own way. Some people may not have a lot of room in the house, or they may not have a garden. For me, going to the park is good because you can walk around, and it is good open space. (A11)*

*When I get out for a walk in the morning, there’s quite a lot of ladies walking, for example, in the park. There’s a lot of ladies out there. (A1)*

However, it was also perceived that adverse weather condition could affect the ability to be active outdoors.

*Here, [ U.K. ] due to weather conditions, you can’t exercise outside. (A10)*.

*If the weather is raining, then I don’t go out. Then I just go up and down in the house (A7)*.

For those who could not undertake EPA in the house or saw barriers to outdoor activities, having access to other facilities such as a community hall was highlighted as a way to facilitate the undertaking of more EPA.

*If you could arrange some activities somewhere that we could go to, to do some classes and make it nice for us to go there in a nice environment and atmosphere. You feel fresh after that and then you’d actually feel like doing the activities as well. (A6)*

However, attending community activities could be challenging unless they had transportation to get them there.

*If somebody takes me and drops me off, then I can go. (A10)*

*Yes, I would [go to exercise classes]. But I haven’t got a car, so how would I be able to go? (A3)*

### Exercise beliefs

Participants’ beliefs about EPA and whether they impacted their physical health seemed to influence whether they were undertaken or not. Those participants who were more motivated to undertake EPA believed that exercises were essential to help them with their physical symptoms as well as their mental wellbeing.

*Yes, [exercise] makes you feel good. Your body gets tired. I feel I used to get a good sleep, otherwise I don’t get much sleep. (A10)*

*Before, I could hardly move my body much. I’ve been told about various physical exercises which have helped me a lot. Slowly, I was able to move my body again. Along with the medicines, and doing the exercises, these have helped a lot (A2)*.

*But I know if I get out of the house and walk it’s good for my mental health too. (A7)*

However, reciprocally, participants’ physical condition influenced their perceived ability to undertake any EPA. Pain and tiredness appeared to be the main symptoms that prevented engagement in EPA.

*When there’s a lot of pain, I just stay upstairs, I don’t come downstairs. I get pain in the joints, knees and feet. And then I stay upstairs mainly. (A1)*

*Sometimes you just don’t even feel like getting out of bed. The movements of the body are so slow. Even if you want to do something, you can’t. Who wants pain in the bones, the body to be slow and you can’t move properly? How can I exercise? (A6)*

*I get tired and my body starts hurting. Sometimes there’s pain in my shoulder, sometimes pain in my hands, knees, back. A lot of the pain, I get in my back (A12)*.

Other participants were deterred from undertaking EPA as they believed doing so would increase their symptoms.

*[I] thought that if I walk, the pain would increase.(A3)*.

*I can’t do it, I have so much pain. Give me some medicine first. You know, give me something to make me better (A8)*.

This view seemed to have been reinforced for participants who had attempted to undertake EPA and felt no relief or, in some cases, an increase in their symptoms.

*During lockdown, I tried to exercise, two or three times a day. Like for instance one day I may have done it in the morning. I may have exercised for an hour. I may have exercised twice in a day. But in lockdown, I did it twice a day continuously. I didn’t feel any benefit. (A12)*

*When I go for a walk, I get a lot of pain in my foot and my leg. I get pains in the veins of my legs. I get a lot of pain at night. (A4)*

### Culture & Language

Some participants seemed to have a stronger belief in their faith and its influence on their condition rather than EPA.

*…It’s my fate… I hope God saves people from this pain and takes away their difficulties and people look after themselves so they don’t feel this pain. (A9)*

*I was quite unwell before but thanks to Allah, I’m much better now. If I can help others, I would like to do that as much as possible. (A2)*

There was a perception that their culture shaped their compatriots’ beliefs about EPA. Many held the view that they were discouraged from undertaking EPA as it was not normal practice for people from their country of birth to engage in it.

*I sometimes get out [in the UK] but in South Asia they tend not to go out. They do a bit of housework, and they get tired doing that. They don’t walk around much. (A7)*

*These Pakistani people don’t understand that exercise is important. They don’t put much effort into it. (A3)*

Participants also highlighted that language differences between them and the health professional affected the ability to understand what they were being told as well as having the capability to clarify their situation.

*When we have hospital appointments, we don’t have the language. I feel I can’t completely explain to the doctor… (A1)*.

*If it’s in your own language, you can get better healthcare support. Language is a very big thing. It’s a big help in your own language. If you say something and I can’t understand you and I’m trying to explain to you, how can I explain to you? (A6)*

There was also a preference, by most, that exercise classes were taught in the same language as theirs so that they could make more sense of it.

*If it’s people from my community that’s fine… If it’s one community and they explain it in one language, then that’s easier (A12).*

*if they explained in Urdu, I would like that (A3).*

## Discussion

This is the first study that has investigated the barriers, enablers and exercise beliefs in non-English speaking SA individuals who have chronic musculoskeletal disease where EPA forms an essential aspect of their chronic pain management [1–4]. Enablers and barriers to EPA were identified among non-English speaking SA people with chronic musculoskeletal disease who were predominantly female and had moderate to severe pain. Participants reported that physical health such as the level of pain and tiredness associated with their chronic musculoskeletal disease, played a significant role in their willingness to engage with EPA. Specifically, several participants expressed fear and reluctance to engage with EPA when experiencing increased pain or feeling tired. Although, EPA has been found to lessen the pain response in chronic musculoskeletal conditions this is only if it is tailored to the individual and their exercise tolerance [37]. Therefore, it is essential to support patient-centred care and the individualisation of EPA to the patients’ needs including considering cultural and communication requirements [38].

We found that culture had a predominant role in shaping the exercise beliefs that subsequently encouraged or discouraged participation in EPA. Several other studies have found EPA is not ingrained within the culture or upbringing of SA communities [22, 39–44] and cultural conflict and acceptability was reported to discourage sport and exercise in women [44–46]. In keeping with previous reports [22, 23, 40, 43, 45], we found cultural nuances as to what EPA may be acceptable and single sex group exercise classes, ideally in their own language, was preferred. However, some disliked the concept of group exercise altogether. Previous work has found that the benefit of group exercise is less evident among people from ethnic minority groups who may feel there is a lack of commonality with the white participants as this limits the exchange of conversation and interaction [22, 47], making attending these classes less enjoyable and more stressful [47].

In line with previous studies which have found lack of language proficiency and low literacy levels negatively impact the ability of SA people to engage with EPA [23, 43, 48–50], language was identified as an important barrier to engagement in the current study, with participants expressing a preference for education in their own language. Previous work in SA populations has identified [23, 51–53] significant difficulty with understanding information discussed and expressing themselves, with Pakistani women in North West England needing a longer time to process the verbal information given and not helped with the constraints of clinic appointment times [23]. The implications are that longer appointment times may be required, particularly when translation services are needed.

We also found language acculturation and literacy could affect knowledge and this supports previous work [23, 43, 48–50]. Knowledge and education play a vital role in the exercise behaviours of SA people who were non-English speaking. This can be seen on two-fronts: the SA individuals not being able to express adequately their need and the support required for engaging in EPA, and clinicians not ascertaining their level of understanding on their know-how to perform EPA. Therefore, when designing a future complex intervention for this subgroup of individuals, improving communication and education to cultivate positive exercise behaviours will be of key importance. Participants should have ready access to health professionals to allow the development of skills-mix and self-exercise efficacy to lead physically active lives, in addition to concerted effort from clinicians to resolve misconceptions or concerns relating to symptom exacerbation from EPA [23, 54–56].

The current study found a number of participants had never had physiotherapy though they may have benefitted from it. Participants expressed a desire to participate in physiotherapy, including learning the skills on how to exercise and manage their symptoms better. Other studies have reported ethnic minority men and women started to engage in EPA after being formally advised to do so by their health physician [40, 41]; for example, Khanam and Costarelli [57] reported 96% of their participants were only willing to take up exercise if they were referred to the gym by their general practitioner as an alternative or additional treatment for their complaints and that they would not exercise voluntarily. Therefore, routine referral to physiotherapy at time of diagnosis of chronic musculoskeletal disease should be encouraged as appropriate.

Having accessibility to EPA class or homes with dedicated areas for exercise or a nearby park were reported to be enablers to EPA. The availability of resources such as free gym access, having a means to travel to or a local exercise class venue, and community-approved exercise programmes were factors that have been previously identified as enablers to EPA [50, 54, 58]. Social prescribing programmes including those promoting exercise for weight reduction and social enjoyment [57] should also cater for this group of individuals via provision of interpreters if native speakers are not available, or via audio and video recordings in SA languages.

Our study found there was an awareness and aptitude towards changing cultural norms and an expression for a more supportive healthcare and social care system. Given that a more positive attitude to EPA has been reported in second generation SA people who were born and brought up in the western countries [40], engaging younger family members could support the promotion of EPA, particularly as inclusion of family and social network has been reported to encourage EPA [54, 58, 59].

Strengths of the study are that, not only is it the first qualitative study to explore the perceptions of symptomatic individuals from SA with chronic musculoskeletal disease where EPA often forms a core treatment, but that it is also the first to focus on the non-English speaking subgroup. The participant age range in the study is reflective of that seen in rheumatology practice meaning our findings may be clinically relevant to other clinicians. However, several limitations must be acknowledged. First, the sample size was modest but comparable to other qualitative studies and recruitment was continued until data saturation was achieved. Second, 10 of 12 participants were of Pakistani origin, meaning that findings may not be generalisable to all people of SA origin; country of origin will impact both the mother-tongue language and cultural aspects. However, the intention of qualitative research is not to produce findings that are generalisable; rather the findings should be transferable and resonate with others. Third, the findings may not be generalisable across other religions as the majority of participants were Islamic. However, Islam is a common religion in ethnic minority groups so we believe the findings are informative for clinical practice. Fourth, the heterogeneity in the conditions of the participants could influence their behaviours; however, the aim of the study was to explore enablers and barriers to engagement with EPA regardless of the rheumatological condition in order to identify common enablers and barriers that could be addressed in a clinical setting. Finally, whilst the study has identified potential areas to address in order to promote engagement with EPA, interventional studies are required to identify the most effective strategies.

## Conclusions

In summary, we found that many of the barriers and enablers to engagement with EPA among SA people with painful chronic musculoskeletal disease were similar to those without these conditions. However, additional interventions are needed to address the level of knowledge on the benefits of EPA in the management of chronic joint and muscle pain. Supporting the development of skills required to exercise safely and confidently, and providing information and services in their first language may also promote the EPA engagement of non-English speaking SA individuals with chronic musculoskeletal disease. The findings should inform future interventions to promote EPA in SA communities with chronic musculoskeletal disease.

## Data Availability

The datasets used and/or analyzed during the current study are available from the corresponding author on reasonable request.

## Abbreviations

EULAR: European League Against Rheumatism
NICE: National Institute for Health and Care Excellence
EPA: exercise and physical activity
SA: South Asian
UK: United Kingdom
NHS: National Health Service
